# Tracking of centriole inheritance in *C. elegans*

**DOI:** 10.17912/micropub.biology.000256

**Published:** 2020-05-28

**Authors:** Anna C. Erpf, Tamara Mikeladze-Dvali

**Affiliations:** 1 Department of Cell and Developmental Biology, Ludwig-Maximilians-University Munich, Grosshaderner Str. 2, 82152 Planegg-Martinsried, Germany; 2 Current address: Lunenfeld-Tanenbaum Research Institute, Sinai Health System, 600 University Avenue, Toronto, ON M5G 1X5, Canada

**Figure 1:**
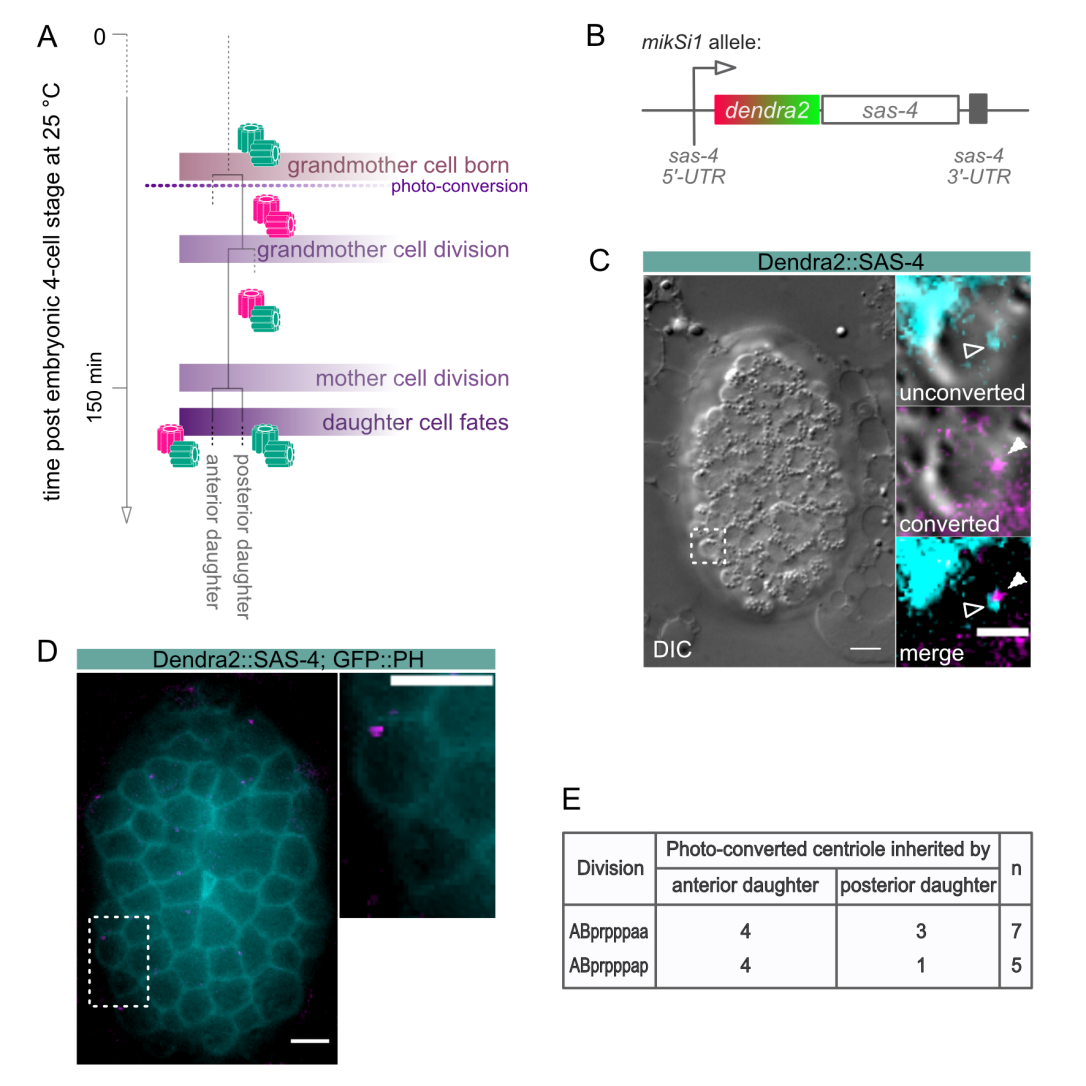
Centrosomes can be successfully tracked within *C. elegans* applying photo-conversion to Dendra2::SAS-4 tagged centrioles. (A) Schematic representation of the UV photo-conversion experiment to analyze the inheritance of Dendra2::SAS-4 tagged centrioles in the ABprpppa cell lineage. Vertical lines indicate the progression of time; horizontal lines indicate cell division events. In this study, grandmother cell refers to: ABprpppa; mother cell: ABprpppaa or ABprpppap; daughter cell: ABprpppaaa, ABprpppaap, ABprpppapa, or ABprpppapp. (B) Schematic representation of the *sas-4p::dendra2::sas-4::sas-4* construct coding for the Dendra2::SAS-4 fusion protein. The *dendra2* coding sequence was fused to the endogenous *sas-4* coding region, flanked by the endogenous *sas-4* regulatory regions. (C) DIC image of a *C. elegans* embryo shortly after the ABprpppaa cell division took place (left panel). The right panel shows DIC and fluorescence images of the anterior daughter cell (ABprpppaaa), which inherited a centrosome comprising a younger unconverted (cyan, white arrow head) and an older converted (magenta, filled white arrow head) centriole. In the merge channel, the young and old centrioles are seen right next to each other as expected for a centrosome before the onset of a new round of centrosome duplication. Scale bar: 5µm, 3µm in the insets. (D) Fluorescence micrographs of a strain expressing Dendra2::SAS-4 and GFP::PH. The expression of GFP::PH allows for clear detection of the cell boundaries and reliable scoring of the segregation of photo-converted centrioles. Images show a photo-converted centriole being inherited by the anterior daughter cell born from the ABprpppaa division. A magenta centriole is present only in the ABprpppaaa cell, but not in the ABprpppaap cell. Only recordings that clearly showed a converted centriole in one daughter cell, but not its sister, were used for the analysis. Scale bars: 5µm. (E) Analysis of age-dependent centriole segregation in the ABprpppaa and ABprpppap cell divisions.

## Description

The nematode *C. elegans* possesses a relatively small subset of centrosome proteins and hence, has emerged as an important model system in elucidating mechanisms of centrosome biogenesis and dynamics. The most basic factors of the centrosome assembly pathway were discovered and characterized in the worm. The centrosome consists of a pair of centrioles, surrounded by the pericentriolar material, and its duplication is strictly coupled to the cell cycle. In worms, the centriole duplication pathway comprises the protein SPD-2^Cep192^, which recruits the kinase ZYG-1^PLK4^ to centrioles to initiate centriole assembly (O’Connell *et al.*, 2001; Kemp *et al.*, 2004; Pelletier *et al.*, 2004). ZYG-1^PLK4 ^in turn, recruits SAS-6^hsSAS6^, which in complex with SAS-5^STIL^ triggers the formation of the central tube (Dammermann *et al.*, 2004; Delattre *et al.*, 2004; Leidel *et al.*, 2005; Kitagawa *et al.*, 2009; Qiao *et al.* 2012; Hilbert **et al.*,* 2013; Lettman *et al.*, 2013; Rogala **et al.*,* 2015). Subsequently, the coiled-coil protein SAS-4^CPAP^ is stably incorporated into the centriole wall and assembles singlet microtubules around the forming centriole (Kirkham *et al.*, 2003; Leidel and Gönczy, 2003, Balestra **et al.*,* 2015). SAS-7^Cep295^ is required for paddlewheel structure formation and recruits SPD-2^Cep192^ for a new round of centriole formation in the following cell cycle (Chang *et al.*, 2016; Saurya *et al.*, 2016; Sugioka *et al.*, 2017). Due to the nature of the duplication of centrioles, there is always one older and one younger centriole present in a centriolar pair. Hence, in the subsequent cell division, daughter cells will inherit centrosomes carrying mother centrioles of different ages. Studies in *Drosophila melanogaster* and mammals have shown that mother centrosome and daughter centrosome can be segregated in a non-random manner during stem cell divisions and that this segregation pattern correlates with the fates of the daughter cells (Yamashita *et al.*, 2007; Wang *et al.*, 2009; Conduit *et al.*, 2010; Januschke *et al.*, 2011). Due to the invariant cell lineage of *C. elegans* and the possibility to follow individual cell fates, tracking centriole inheritance in regard to their age in worms could provide valuable information about the impact of centriole age on differentiation (Sulston and Schierenberg, 1983). However, factors that localize to one of the two centrioles in an age-dependent manner have not been identified in *C. elegans* to date, making it impossible to distinguish older from younger centrosomes in worms. To overcome the limitation of following age-related centrosome inheritance in *C. elegans*, we generated a strain in which centrioles are labeled with a photo-switchable marker. Once centrioles are photo-converted, they can be tracked over several cell cycles, and centrosome age can be distinguished after the second round of duplication (Figure 1A). The photo-switchable fluorescent protein Dendra originates from the octocoral *Dendronephthya sp.* The protein can be irreversibly converted from a green-to-red fluorescence state by exposure to visible blue or ultraviolet light. In this study, we made use of the bright and fast-maturing Dendra2 version of the protein, which was fused to the centriolar protein SAS-4^CPAP^ and expressed under endogenous regulatory sequences (Figure 1 B, Gurskaya *et al.*, 2006, Ihara *et al.*, 2011). SAS-4^CPAP^ is stably incorporated into centrioles and shows no cytoplasmic exchange once centrioles are formed (Dammermann *et al.*, 2004, Balestra **et al.*,* 2015). We did not notice any significant difference in embryonic lethality of the strain at 25°C. On average we found 1.4% embryonic lethality for *sas-4p::dendra2::sas-4* (n=1322 embryos); in comparison to 1.1% for a *wild-type* control strain (n=1692 embryos). In this study, we show that the older and younger centrosome can be successfully distinguished within cells of different lineages in *C. elegans* (Figure 1 C).

L4 larvae were grown at 25°C overnight to adulthood. The next day, worms were dissected to collect embryos shortly after fertilization in H_2_O on a coverslip (Carl Roth GmbH; 18 x 18 mm, #1 thickness; Cat. no. 0657.2). Embryos were reversely mounted on a 4 % agarose pad on a microscope slide and sealed with petroleum jelly. The development of embryos starting at 4-cell stage was followed under a 4D-microscope at 25°C. The lineaging of the cells of interest was performed simultaneously while recording. Images were taken using a Zeiss Axio Imager.M2 equipped with epifluorescence and the Time to Live software from Caenotec. Differential interference contrast (DIC) micrograph Z-stacks were taken every 35 sec at 25°C. Fluorescent scans were taken as required. The Simi BioCell software was used for the lineage analysis (Simi Reality Motion Systems GmbH; http://www.simi.com) as previously described (Schnabel *et al.*, 1997). Embryos were allowed to develop until shortly after the onset of the ABprppp cell division. Subsequently, embryos were exposed to UV light on a Zeiss Axioscop 2 microscope for photo-conversion of the Dendra2 fluorophore (conversion time: 15-17 sec; whole embryos were exposed to UV light, and all centrioles present at this stage were photo-converted). All centrioles formed prior to photo-conversion display red fluorescence (magenta) after the exposure to UV light, whereas centrioles formed after conversion display green fluorescence (cyan; Figure 1 A). Lineaging of the embryos was continued on the 4D microscope after photo-conversion.

As proof of principle, we analyzed the age-dependent inheritance pattern of centrioles in a strain expressing Dendra2::SAS-4, as well as GFP::PH, to reliably mark the outlines of individual cells. The cell lineage chosen was still easy to track but close to the final differentiation state. Centrioles were photo-converted in the grandmother cell ABprpppa (Figure 1 A). The divisions of the daughter cells ABprpppaa and ABprpppap were followed to determine the segregation of the centrioles (Figure 1 D and E). Fluorescence images were taken after the divisions of the sister cells. The ABprpppaa daughter cell divides into equally sized granddaughter cells. Here, in 57 % of the divisions, the older converted centriole is inherited by the anterior cell, and in 43 % of the divisions by the posterior cell (n=7, Figure 1 E). The granddaughter cells deriving from the ABprpppap cell division are unequal in size, with the posterior granddaughter being smaller and fated to die. In 80 % of the divisions, the older converted centriole segregates into the anterior cell. In the remaining 20 % of the cases, the posterior cell inherits the converted centriole (n=5, Figure 1 E). Thus, despite what fate the granddaughter cells adapt, centrioles are segregated randomly in both lineages. Taken together, our approach allows the successful tracking of centrosome inheritance in the invariant divisions of the *C. elegans* lineage.

## Methods

We applied standard methods for DNA amplification, analysis, and manipulation. For PCR amplification, the Phusion® High-Fidelity DNA Polymerase (New England Biolabs) was used according to the manufacturer’s protocol. The *sas-4* sequence and regulatory regions were amplified from *C. elegans* genomic DNA and pAD154. The *dendra2* nucleotide sequence was introduced upstream of the *sas-4* coding sequence in the MosSCI vector pCFJ350 to generate the TMD29 [*sas-4p::dendra2::sas-4::sas-4*] plasmid (Figure 1 B). Cloning was performed via the sequence and ligation independent cloning (SLIC) method (Jeong *et al.*, 2012) using the T4 DNA polymerase (New England Biolabs) and NEBuffer 2.1 (New England Biolabs). Sanger sequencing was applied to analyze DNA sequences. The TMD29 plasmid was integrated into the second chromosome of the *C. elegans* genome by the universal MosSCI single-copy integration method (Frøkjær-Jensen *et al.*, 2014, integration strain: EG6699 [*ttTi5605; unc-119(ed3)*]) to generate the *mikSi1* allele. Germline microinjection was performed as described by Mello *et al.*. (1991) (Mello and Kramer, 1991). Image analyses were performed with Fiji/ImageJ 2.0.0 (Schindelin *et al.*, 2012, https://fiji.sc/). To test for embryonic lethality, singled L4 worms were allowed to lay eggs at 25°C overnight. Total embryos laid and hatched worms were scored in three independent experiments. The N2 Bristol strain was used as control.

## Reagents

Nematode strains:

N2 *C. elegans* wild isolate

TMD42 *mikSi1[sas-4p::dendra2::sas-4]II; unc-119(ed3)III*

TMD57 *mikSi1[sas-4p::dendra2::sas-4]II; bcIs57*[*pie-1p::gfp::plcδph*]

Nematode strains were maintained at 15 °C under standard conditions (Brenner, 1974).

TMD57 will be made available on CGC.

## References

[R1] Balestra FR, von Tobel L, Gönczy P (2015). Paternally contributed centrioles exhibit exceptional persistence in C. elegans embryos.. Cell Res.

[R2] Brenner S (1974). The genetics of Caenorhabditis elegans.. Genetics.

[R3] Chang CW, Hsu WB, Tsai JJ, Tang CJ, Tang TK (2016). CEP295 interacts with microtubules and is required for centriole elongation.. J Cell Sci.

[R4] Conduit PT, Brunk K, Dobbelaere J, Dix CI, Lucas EP, Raff JW (2010). Centrioles regulate centrosome size by controlling the rate of Cnn incorporation into the PCM.. Curr Biol.

[R5] Dammermann A, Müller-Reichert T, Pelletier L, Habermann B, Desai A, Oegema K (2004). Centriole assembly requires both centriolar and pericentriolar material proteins.. Dev Cell.

[R6] Delattre M, Leidel S, Wani K, Baumer K, Bamat J, Schnabel H, Feichtinger R, Schnabel R, Gönczy P (2004). Centriolar SAS-5 is required for centrosome duplication in C. elegans.. Nat Cell Biol.

[R7] Frøkjær-Jensen C, Davis MW, Sarov M, Taylor J, Flibotte S, LaBella M, Pozniakovsky A, Moerman DG, Jorgensen EM (2014). Random and targeted transgene insertion in Caenorhabditis elegans using a modified Mos1 transposon.. Nat Methods.

[R8] Gurskaya NG, Verkhusha VV, Shcheglov AS, Staroverov DB, Chepurnykh TV, Fradkov AF, Lukyanov S, Lukyanov KA (2006). Engineering of a monomeric green-to-red photoactivatable fluorescent protein induced by blue light.. Nat Biotechnol.

[R9] Hilbert M, Erat MC, Hachet V, Guichard P, Blank ID, Flückiger I, Slater L, Lowe ED, Hatzopoulos GN, Steinmetz MO, Gönczy P, Vakonakis I (2013). Caenorhabditis elegans centriolar protein SAS-6 forms a spiral that is consistent with imparting a ninefold symmetry.. Proc Natl Acad Sci U S A.

[R10] Ihara S, Hagedorn EJ, Morrissey MA, Chi Q, Motegi F, Kramer JM, Sherwood DR (2011). Basement membrane sliding and targeted adhesion remodels tissue boundaries during uterine-vulval attachment in Caenorhabditis elegans.. Nat Cell Biol.

[R11] Januschke J, Llamazares S, Reina J, Gonzalez C (2011). Drosophila neuroblasts retain the daughter centrosome.. Nat Commun.

[R12] Jeong JY, Yim HS, Ryu JY, Lee HS, Lee JH, Seen DS, Kang SG (2012). One-step sequence- and ligation-independent cloning as a rapid and versatile cloning method for functional genomics studies.. Appl Environ Microbiol.

[R13] Kemp CA, Kopish KR, Zipperlen P, Ahringer J, O'Connell KF (2004). Centrosome maturation and duplication in C. elegans require the coiled-coil protein SPD-2.. Dev Cell.

[R14] Kirkham M, Müller-Reichert T, Oegema K, Grill S, Hyman AA (2003). SAS-4 is a C. elegans centriolar protein that controls centrosome size.. Cell.

[R15] Kitagawa D, Busso C, Flückiger I, Gönczy P (2009). Phosphorylation of SAS-6 by ZYG-1 is critical for centriole formation in C. elegans embryos.. Dev Cell.

[R16] Leidel S, Delattre M, Cerutti L, Baumer K, Gönczy P (2005). SAS-6 defines a protein family required for centrosome duplication in C. elegans and in human cells.. Nat Cell Biol.

[R17] Leidel S, Gönczy P (2003). SAS-4 is essential for centrosome duplication in C elegans and is recruited to daughter centrioles once per cell cycle.. Dev Cell.

[R18] Lettman MM, Wong YL, Viscardi V, Niessen S, Chen SH, Shiau AK, Zhou H, Desai A, Oegema K (2013). Direct binding of SAS-6 to ZYG-1 recruits SAS-6 to the mother centriole for cartwheel assembly.. Dev Cell.

[R19] Mello CC, Kramer JM, Stinchcomb D, Ambros V (1991). Efficient gene transfer in C.elegans: extrachromosomal maintenance and integration of transforming sequences.. EMBO J.

[R20] O'Connell KF, Caron C, Kopish KR, Hurd DD, Kemphues KJ, Li Y, White JG (2001). The C. elegans zyg-1 gene encodes a regulator of centrosome duplication with distinct maternal and paternal roles in the embryo.. Cell.

[R21] Pelletier L, Ozlü N, Hannak E, Cowan C, Habermann B, Ruer M, Müller-Reichert T, Hyman AA (2004). The Caenorhabditis elegans centrosomal protein SPD-2 is required for both pericentriolar material recruitment and centriole duplication.. Curr Biol.

[R22] Qiao R, Cabral G, Lettman MM, Dammermann A, Dong G (2012). SAS-6 coiled-coil structure and interaction with SAS-5 suggest a regulatory mechanism in C. elegans centriole assembly.. EMBO J.

[R23] Rogala KB, Dynes NJ, Hatzopoulos GN, Yan J, Pong SK, Robinson CV, Deane CM, Gönczy P, Vakonakis I (2015). The Caenorhabditis elegans protein SAS-5 forms large oligomeric assemblies critical for centriole formation.. Elife.

[R24] Saurya S, Roque H, Novak ZA, Wainman A, Aydogan MG, Volanakis A, Sieber B, Pinto DM, Raff JW (2016). Drosophila Ana1 is required for centrosome assembly and centriole elongation.. J Cell Sci.

[R25] Schindelin J, Arganda-Carreras I, Frise E, Kaynig V, Longair M, Pietzsch T, Preibisch S, Rueden C, Saalfeld S, Schmid B, Tinevez JY, White DJ, Hartenstein V, Eliceiri K, Tomancak P, Cardona A (2012). Fiji: an open-source platform for biological-image analysis.. Nat Methods.

[R26] Schnabel R, Hutter H, Moerman D, Schnabel H (1997). Assessing normal embryogenesis in Caenorhabditis elegans using a 4D microscope: variability of development and regional specification.. Dev Biol.

[R27] Sugioka K, Hamill DR, Lowry JB, McNeely ME, Enrick M, Richter AC, Kiebler LE, Priess JR, Bowerman B (2017). Centriolar SAS-7 acts upstream of SPD-2 to regulate centriole assembly and pericentriolar material formation.. Elife.

[R28] Sulston JE, Schierenberg E, White JG, Thomson JN (1983). The embryonic cell lineage of the nematode Caenorhabditis elegans.. Dev Biol.

[R29] Wang X, Tsai JW, Imai JH, Lian WN, Vallee RB, Shi SH (2009). Asymmetric centrosome inheritance maintains neural progenitors in the neocortex.. Nature.

[R30] Yamashita YM, Mahowald AP, Perlin JR, Fuller MT (2007). Asymmetric inheritance of mother versus daughter centrosome in stem cell division.. Science.

